# Revisiting the origin of electrochemical activity in the topological semimetal PtGa

**DOI:** 10.1039/d6sc03026b

**Published:** 2026-06-11

**Authors:** Brandon Johnston, Meng-Fu Chen, Mingxuan Zhu, Peng Guo, Su-Yang Xu, Joonho Lee, Daniel G. Nocera

**Affiliations:** a Department of Chemistry and Chemical Biology, Harvard University 12 Oxford St. Cambridge 02138 MA USA suyangxu@fas.harvard.edu joonholee@g.harvard.edu dnocera@fas.harvard.edu

## Abstract

Topological materials have been reported as active and selective electrochemical catalysts for a variety of small-molecule energy-conversion reactions. The exceptional activity and selectivity of these materials have been attributed to their topologically non-trivial surface states, which support chiral spin currents and boast high carrier mobilities. For the topological semimetal PtGa, we find that these states are not robust under practical electrochemical conditions. During electrochemical operation, Ga rapidly corrodes from the surface, yielding a nanoporous, Pt-rich layer. Inductively coupled plasma mass spectrometry and scanning transmission electron microscopy imaging independently confirm Ga corrosion. Hydrogen evolution reaction activity measurements demonstrate that the electrocatalytic performance of PtGa is correlated with the number of Pt active sites, and first-principle calculations further show that introducing Ga vacancies into the PtGa crystal structure disrupts the topologically non-trivial surface states. These observations suggest that previous reports detailing the high electrochemical activity of PtGa might be better explained by an enrichment in Pt active sites following corrosion, rather than by a genuine increase in the intrinsic reaction rate mediated by PtGa's topological surface states. These results urge caution in attributing enhanced catalytic activity to topological surface states without verifying surface composition and electronic structure. They also highlight the distinct meaning of robustness in physics and chemistry.

## Abstract 2

The electrochemical activity of PtGa arises from Ga corrosion that enriches surface Pt sites, instead of previously claimed topological surface states.

## Graphical abstract

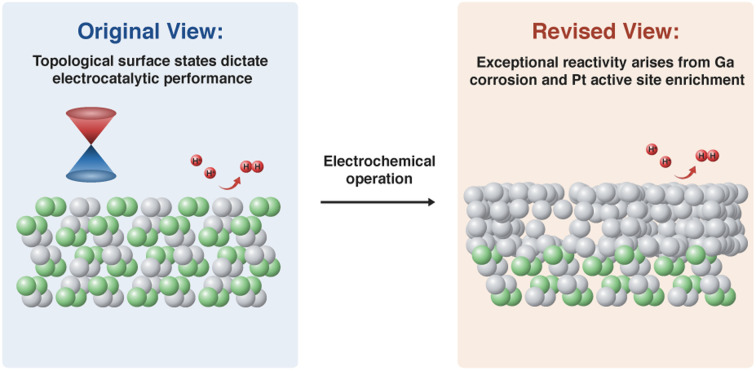

## Introduction

Topological materials have attracted significant attention in condensed matter physics. Notably, they have an inverted band structure that leads to the formation of robust, topologically-protected surface states,^1^ which are electronically conductive and support chiral spin currents.^2^ The nontrivial topology, robust surface state, highly metallic surface conduction and chiral spin textures have led to emerging interest in exploring topological materials as heterogeneous catalysts for electrochemical reactions,^3–8^ as they may promote unique electrochemical reaction selectivity^9–13^ and faster intrinsic reaction rates. Topological semimetals (TSMs) represent perhaps the most intriguing class of candidate electrocatalysts, as they boast high carrier mobilities^14^ and large topologically non-trivial energy windows around the Fermi level.^15,16^ Notably, many TSMs have topological surface states derived from d-orbitals, which are known to form strong bonding interactions with adsorbates and play a prominent role in dictating transition metal catalytic reactivity.^17,18^ Particular attention has been paid to the activity of TSMs towards the hydrogen evolution reaction (HER), with previous reports indicating that transition metal monosilicides (MSi)^5^ and Pt-based TSMs such as PtSn_4_,^3^ PtAl, and PtGa^13^ boast high activity that rival state-of-the-art Pt electrocatalysts.

In most cases, experimental evidence supporting these claims have come in the form of current–potential curves demonstrating high current densities at low reaction overpotentials. However, this does not necessarily reflect higher intrinsic catalytic activity. As shown in eqn (1),^19^ the current density (*j*) of an electrocatalytic reaction is related to the intrinsic reaction rate (*k*^0^), the concentration of active sites (*C*_as_), and the reaction overpotential (*η*). Additional parameters in eqn (1) include Faraday's constant (*F*), the universal gas constant (*R*), the reaction temperature (*T*), the charge transfer coefficient (*α*), and the number of electrons involved in the electrochemical process (*n*).1
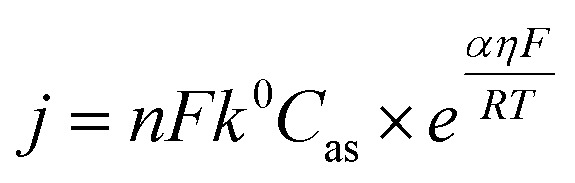


Thus, in cases where the crystalline form of the catalyst is corroded or amorphized under electrochemical conditions, the reaction current density may increase due to an increase in new active sites (*C*_as_) rather than from changes in the intrinsic reaction kinetics (*k*^0^).^20–22^ Therefore, it is imperative to evaluate the stability and structural evolution of heterogeneous electrocatalysts under operating conditions.^23,24^ To be practically implemented as catalysts for electrochemical reactions, topological materials must therefore have robust topological surface states under external perturbation.^25^ It has been shown both experimentally and computationally that topological surface states are preserved under surface atom adsorption,^26^ deposition of thin metallic films on the substrate,^27,28^ and exposure to ambient atmospheric conditions.^29^ However, these perturbations do not reflect the *operando* electrochemical environment, which often involves strongly oxidizing or reducing conditions and highly acidic or basic environments. Stability in these environments, which is necessary for practical implementation in electrochemical devices, has not been thoroughly explored for topological materials. Recent theoretical work has studied the evolution of topological surface states under realistic electrochemical reaction conditions.^30–34^ For example, the surface state evolution of PtSn_4_ during HER catalysis was carefully studied using first-principles calculations.^32^ However, studies that experimentally monitor the surface evolution of topological materials under electrochemical reaction conditions remain underexplored.

To this end, we became particularly interested in exploring the stability of PtGa, a TSM that has been proposed to catalyze the HER with higher H_2_ turnover frequencies than Pt metal in acidic media.^13^ The improved activity of PtGa was attributed to the catalyst's topological band structure, which may promote rapid H_2_ desorption kinetics by lowering the binding energy of surface-adsorbed hydrogen. Similar catalytic enhancements have been observed for the oxygen reduction reaction (ORR), as well as improved selectivity for water as the terminal product *via* proposed catalyst spin polarization effects.^10^ The exceptional activity of PtGa is particularly interesting because Pt, which is widely considered a privileged catalyst for both the HER and ORR, is expensive to mine and source.^35^ Therefore, there is significant research interest in developing Pt-based electrocatalysts that minimize precious metal loading by incorporating inexpensive and earth-abundant metals and metalloids,^36–38^ such as Ga.^39–42^ However, non-precious metals undergo facile corrosion in aqueous conditions,^43^ which has been intentionally leveraged to generate porous dealloyed materials.^44–47^ Thus, verification of PtGa's structural stability under electrochemical conditions is crucial for clearly establishing a relationship between the catalyst's predicted surface states and its observed electrochemical performance.

Herein, we show that the crystalline structure of PtGa is not robust in an electrochemical working environment. Using a combination of electrochemical, mass spectrometry, and electron microscopy techniques, we show that PtGa during electrochemical operation corrodes and leaches Ga in acidic media, leading to the formation of a nanoporous layer of a Pt-enriched phase at the electrode surface. We further present computational results that show the loss of topological character in PtGa surface bands following Ga atom corrosion. On the basis of these combined experimental and computational pieces of evidence, we propose that the exceptional electrochemical performance of PtGa for electrochemical H_2_ generation results from an increase in the concentration of Pt active sites (*C*_as_) during Ga corrosion rather than an increase in the intrinsic reaction rate (*k*^0^) mediated by PtGa's topological band structure. This work highlights the critical importance of structural analysis and active site quantification for accurately benchmarking the intrinsic electrocatalytic activity of topological materials.

## Results

### PtGa crystal synthesis and characterization

PtGa ingots were synthesized using a previously described flux technique.^48^ Consistent with these reports, PtGa crystallizes in the chiral *P*2_1_3 space group with lattice parameter *a* = 4.8979(3) Å (SI Fig. S1 and Table S1). Under scanning electron microscopy (SEM), large single crystalline domains are observed (SI Fig. S10a), the elemental composition of which are 49(1)% Pt and 51(1)% Ga, as measured by energy-dispersive X-ray spectroscopy (EDS). Electron backscatter diffraction (EBSD) analysis (SI Fig. S10b) shows that the Kikuchi pattern of these domains index to the crystal structure of PtGa and that their crystalline orientation is confined to a very small range in orientation space (SI Fig. S10c). We note that the entire ingot is not single crystalline, with minor contributions from regions containing 61(1)% Pt and 39(1)% Ga, as measured by EDS. The surface composition was further evaluated using X-ray photoelectron spectroscopy (XPS). As shown in SI Fig. S2a, the Pt 4f XPS spectra of PtGa show two peaks with binding energies of 71.8 eV and 75.1 eV. These peaks correspond to the Pt 4f_7/2_ and Pt 4f_5/2_ peaks, respectively, and match previously reported Pt 4f binding energies for single crystal PtGa.^10^ The corresponding Ga 3d spectra show a single peak at 19.4 eV that arises from a convolution of the Ga 3d_5/2_ and Ga 3d_3/2_ peaks at 19.3 and 19.7 eV, respectively (SI Fig. S2b). No shifts or changes in intensity are observed in the Pt 4f (SI Fig. S2c) or Ga 3d (SI Fig. S2d) XPS spectral regions upon Ar sputter-cleaning, demonstrating that the PtGa crystal surface is compositionally identical to the bulk prior to electrochemical operation.

### Electrochemical activity of PtGa

**Fig. 1:**
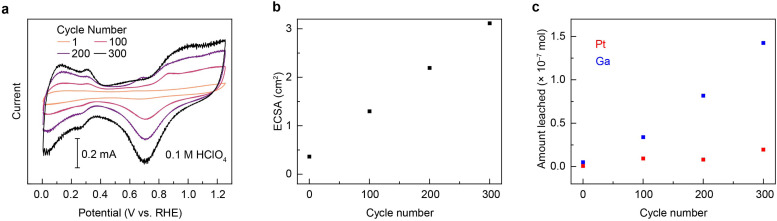
Electrochemical activity of PtGa. (a) CVs of PtGa (scan rate = 50 mV s^−1^). Additional parameters provided in figure legend. (b) ECSA of PtGa during electrochemical operation, as determined by CO stripping voltammetry. (c) Amount of Ga and Pt in the electrolyte solution during electrochemical operation, as determined by ICP-MS.

Cyclic voltammograms (CV) of PtGa in 0.1 M HClO_4_ (pH = 1.00), cycled in a non-faradaic region, are shown in Fig. 1a. All electrochemical experiments were performed in acid to best match the conditions used in previous reports detailing the electrochemical activity of single crystal PtGa. The voltammograms change dramatically with increasing cycle number, unveiling well-defined hydrogen adsorption and desorption peaks at 0.25 V and 0 V *vs.* RHE, respectively. These peaks match exactly those observed for pseudocapacitive hydrogen adsorption to Pt active sites, which are widely used to determine electrochemical active surface area (ECSA).^49^ Thus, the growth of these adsorption and desorption features are consistent with an increase in the number of Pt active sites during electrochemical operation. The increasing number of Pt active sites was separately confirmed and quantified using electrochemical CO stripping (SI Fig. S3). After 300 cycles, the ECSA has increased by a factor of 10 (Fig. 1b), and the corresponding voltammogram (Fig. 1a, black trace) matches that of a Pt disk cycled in the same electrochemical window (SI Fig. S4). Inductively coupled plasma mass spectrometry (ICP-MS) analysis reveals that the concentration of Ga and Pt in the electrolyte solution increases during electrochemical operation (Fig. 1c), suggesting that Ga and Pt both leach from the PtGa crystal lattice. However, Ga leaches more readily, at a roughly 8 : 1 ratio relative to Pt.

To better understand how Ga corrosion affects the electrocatalytic performance of PtGa, HER activity measurements (*E*_appl_ = −0.02 V *vs.* RHE) were performed before and after electrochemical cycling between 0.0 V and 1.2 V *vs.* RHE in 0.1 M HClO_4_ (pH = 1.00). As shown in SI Fig. S5, the measured HER current density, when normalized by the apparent geometric surface area, increases as PtGa is cycled electrochemically. In fact, after 300 cycles, the apparent HER current density is twice the initial current density. However, when the current density is normalized to the ECSA determined by electrochemical CO stripping, the current density decreases slightly with cycle number. This suggests that the increase in apparent HER current density arises from the exposure of new active sites following Ga corrosion, consistent with the data shown in Fig. 1b. Moreover, the ECSA normalized HER current density of freshly polished PtGa is lower than that observed for Pt, indicating that PtGa is an inferior HER catalyst.

### Electron microscopy imaging of PtGa following electrochemical operation

**Fig. 2:**
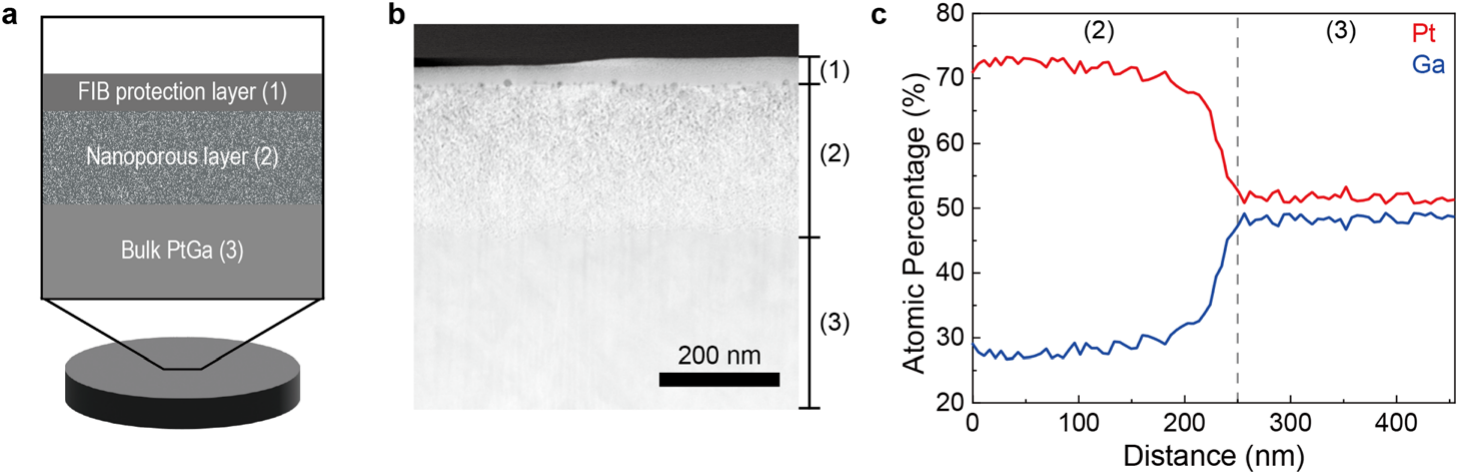
Electron microscopy analysis of PtGa. (a) Schematic of the lamella prepared by FIB from the region that underwent electrochemical treatment. (b) Annular dark-field (ADF) STEM image of the lamella showing the (1), (2) and (3) regions illustrated in the schematic. (c) EDS analysis of the lamella. Distance means the distance from the original PtGa surface, which is the boundary of layers (1) and (2).

To further analyze the thickness, morphology, and elemental composition of the PtGa crystal following electrochemical operation, a lamella of the surface (Fig. 2a) was prepared by focused ion beam (FIB) microscopy (SI Fig. S6). Before FIB milling, carbon and tungsten protection layers were deposited on the surface to prevent any surface damage during FIB milling. After the lamella was prepared, its sidewalls were polished by Ar plasma to remove residual Ga implantation from FIB milling.^50^ The lamella was then analyzed by scanning transmission electron microscopy (STEM). A nanoporous layer with morphology that differs significantly from the bulk is observed to extend 250 nm beneath the surface (Fig. 2b and S8) following 800 CV cycles between 0.0 and 1.2 V *vs.* RHE. This nanoporous layer is absent from the lamella prepared from the same PtGa crystal prior to electrochemical operation (SI Fig. S7). The pores near the surface are slightly larger than those beneath, and the boundary between the nanoporous layer and the bulk is sharp and well defined. EDS was performed on the lamella to determine the elemental composition of each layer. The average Pt and Ga content in the top 200 nm of the nanoporous layer are 72(1)% and 28(1)%, respectively. In the bulk crystal, the Pt and Ga content are 52(1)% and 48(1)%, respectively, which agrees with the results from single crystal X-ray diffraction (SCXRD) analysis (SI Fig. S1). These findings corroborate the electrochemical and ICP results, which show that Ga leaches from the crystal during electrochemical operation, leaving behind a Pt-enriched nanoporous layer near the electrode surface.

The results described above clearly establish that PtGa leaches Ga when electrochemically cycled between 0.0 V and 1.2 V *vs.* RHE (pH = 1.00). To further evaluate the electrochemical stability of PtGa under more reducing conditions, chronoamperometry was performed at −0.09 V *vs.* RHE in 0.1 M HClO_4_ (pH = 1.00). Active site concentration determination by CO stripping was not performed to avoid scanning to more oxidizing potentials where Ga leaching occurs. ICP-MS analysis following operation reveals that Ga leaches into solution (1.2 × 10^−8^ mol), although at a much slower rate than when PtGa is electrochemically cycled between 0.0 V and 1.2 V *vs.* RHE. As before, a lamella of the PtGa crystal surface was prepared by FIB milling. Unlike the lamella prepared following electrochemical cycling between 0.0 V and 1.2 V *vs.* RHE (Fig. 2b), a nanoporous layer that extends deep below the surface is not observed by STEM imaging (SI Fig. S9). Instead, a nanoporous layer 10 nm in thickness, which is absent from the lamella prepared prior to electrochemical operation, is observed.

### Effect of Ga vacancy formation on topological band structure from *ab initio* calculations

**Fig. 3:**
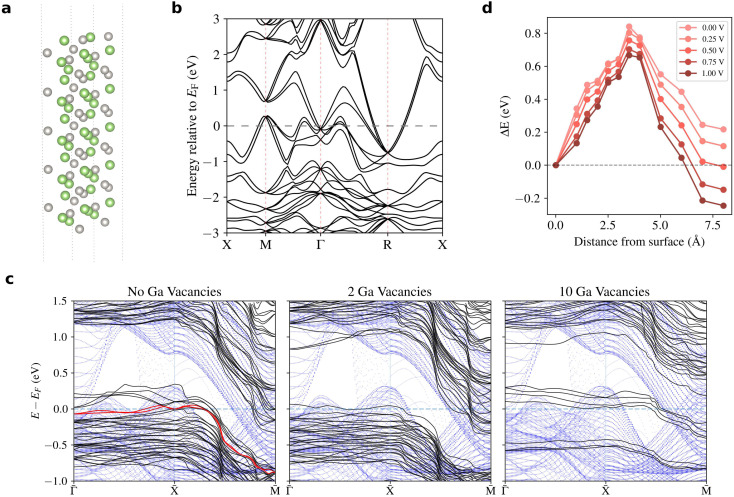
(a) Schematic of PtGa (001) slab model. A slab of five-unit cell thickness was used in our calculations. (b) DFT band structure of bulk PtGa with spin–orbit coupling. (c) Comparison of the (001) surface band structure between the pristine PtGa slab and the one with vacancies introduced. The black curves show the states from the top surface. The bulk states projected onto the (001) surface are shown as blue curves in the background. (d) Dissociation energy curve of the Ga atom under different oxidative potentials.

To investigate the effect of Ga atom corrosion on the band structure and topological surface states of PtGa, we carried out density functional theory (DFT) calculations to obtain the band structures of the PtGa crystal before and after Ga corrosion. To carry out surface band calculations, we used a slab model of the (001) facet of PtGa, as shown in Fig. 3a. To simulate the crystal structure after Ga leaching, we removed Ga atoms from the top layers of the slab to model Ga corrosion and subsequent vacancy formation. The number of Ga atoms removed from the slab was gradually increased until the experimentally observed ratio was reached, and the crystal structure was allowed to relax to most closely mimic a possible experimental structure. More details on the calculation setup can be found in the SI.

To validate our computational setup, we first performed band-structure calculations with spin–orbit coupling for the PtGa bulk crystal and obtained the DFT band structure shown in Fig. 3b. One can see that the four-fold degenerate point at *Γ* and the six-fold degenerate point at *R*, both with non-zero Chern numbers, can be observed, consistent with previous computational^13,48^ and experimentally obtained angle-resolved photoemission spectroscopy (ARPES) results.^48^ We then performed calculations for a pristine (001) slab, a slab with the Ga atoms of the top layer removed (2 Ga vacancies), and a slab with a composition that matches the experimentally observed Pt : Ga ratio following electrochemical operation (10 Ga vacancies). In Fig. 3c, we compare the bands that are localized on the top layer of the slab (plotted in black) with a background of the bulk states projected onto the (001) surface (plotted in blue). In the pristine slab, one can see the bands (shown in red) which originate and end at the high degeneracy Weyl nodes at the projected *Γ̄* point and *M̄* points and cross the Fermi level, which are signatures of the Fermi arcs in this material. However, these characteristic bands are lost when 2 Ga vacancies are introduced in the top layer of the slab, and is even more strongly modified when more vacancies are introduced, which is accompanied by significant lattice reconstruction.

To further understand the Ga dissociation process at the surface of PtGa, we carried out grand-canonical density functional theory (GC-DFT) calculations to simulate the electrical bias-dependent free energy profile of the dissociation process.^51–54^ In this process, the electron number in the system is allowed to vary until the Fermi level of the system reaches the desired oxidative potential relative to the computational hydrogen electrode, which more closely mimics the environment of electrochemical cells compared to traditional fixed-electron number DFT. This approach has previously been used to study other corrosion processes, such as silver electrodissolution.^55^ In Fig. 3d, we show the bias-dependent dissociation energy profiles for Ga dissolution from the PtGa slab. We can see that the dissociated state becomes increasingly thermodynamically favorable at more positive oxidative potentials, with the associated kinetic barrier also lowered relative to that at 0.0 V *vs.* RHE. This result qualitatively agrees with the experimental observation of increased Ga corrosion rates at oxidative potentials and further supports our observation of Ga atom leaching from the PtGa crystal under electrochemical operating conditions.

## Discussion

We began by investigating the electrochemical stability of PtGa in an aqueous non-faradaic potential window (0.0 V to 1.2 V *vs.* RHE). Stability in this potential regime is crucial for the practical implementation of PtGa as a heterogeneous electrocatalyst in aqueous conditions. As shown in Fig. 1b and S3, the ECSA of the crystal increases dramatically when electrochemically cycled between 0.0 V and 1.2 V *vs.* RHE. This increase in surface area is clearly correlated with Ga leaching from the electrode surface (Fig. 1c), demonstrating that PtGa corrodes under practical electrochemical conditions. The ADF-STEM cross-section images of the PtGa crystal following electrochemical operation show that the surface becomes nanoporous (Fig. 2b) and highly enriched in Pt (Fig. 2c). After 800 electrochemical cycles, the nanoporous layer extends 250 nm beneath the surface. Assuming a linear growth rate, this suggests the nanoporous layer grows by 0.31 nm per cycle. Gratifyingly, this value is consistent with the ICP-MS data shown in Fig. 1. Assuming the same growth rate, the nanoporous layer would be expected to extend 93 nm beneath the surface after 300 cycles. Using the crystal's lattice constant (4.8979 Å), the ECSA at the beginning of the electrochemical experiment (0.36 cm^2^), and the average stoichiometry of the nanoporous layer from STEM-EDS (Fig. 2c), we find the expected moles of Ga leached to be 1.2 × 10^−7^ mol (see SI for detailed calculation methodology), which agrees well with the value observed from ICP-MS data (1.4 × 10^−7^ mol). Remarkably, a leaching rate of 0.31 nm per cycle would imply that an entire unit cell at the electrode/electrolyte interface could be destroyed after only two electrochemical cycles. This suggests that even performing ECSA measurements, such as electrochemical CO stripping, which require scanning to oxidizing potentials (CO oxidation occurs at 0.7 V *vs.* RHE), is sufficient to damage the crystal structure at the electrode surface. Therefore, benchmarking the intrinsic catalytic activity of PtGa requires rigorous active site quantification. As shown in SI Fig. S5, when the HER activity of PtGa and Pt are normalized to the number of Pt active sites using CO stripping voltammetry, lower current densities are observed for PtGa, in contrast to previous reports. Larger apparent current densities can be observed by inducing Ga corrosion *via* electrochemical cycling, but these increases arise from an increase in ECSA, rather than from a genuine increase in catalytic activity. These results suggest that the exceptional activity of PtGa towards the HER does not arise from PtGa's topological surface states. Instead, PtGa's exceptional activity might be attributed to an increase in the density of Pt active sites (eqn (1)) following the formation of a nanoporous, Pt-enriched structure near the electrode surface. Accounting for this increase in active site concentration would be challenging because it occurs during electrochemical operation, and it would result in a larger observed reaction current density (*j*) at a particular overpotential (*η*) (see eqn (1)). Additionally, since the ORR occurs under the potential window where rapid Ga corrosion was observed,^10^ similar Ga corrosion may be expected under ORR-relevant conditions.

The oxidative instability of PtGa in acidic media can be easily rationalized using Ga's Pourbaix diagram.^43^ At pH = 1.00, Ga oxidation to Ga^3+^ becomes thermodynamically favorable at potentials more positive than −0.529 V *vs.* SHE. Thus, Ga corrosion might be expected even under conditions that are sufficiently reducing to evolve hydrogen. When the electrode potential is maintained at −0.09 V *vs.* RHE, Ga leaching (1.2 × 10^−8^ mol) is still observed. The corresponding STEM imaging results (SI Fig. S9) show the formation of a nanoporous layer extending 10 nm beneath the surface, but the relative concentrations of Pt and Ga from EDS mapping match that seen in the bulk. To explain this result, we attribute the formation of the nanoporous layer to structural damage caused by hydrogen evolution. Still, the formation of a nanoporous layer and the observation of Ga corrosion by ICP-MS have important implications for HER catalysis, though the extent of Ga leaching is less dramatic than observed following electrochemical cycling between 0.0 V and 1.2 V *vs.* RHE. Thus, even under more reducing conditions the observed electrocatalytic activity of PtGa towards HER might be more readily explained by Ga corrosion and Pt active site enrichment. This may be especially true if HER experiments are conducted following ECSA measurements that require scanning to more oxidizing potentials where rapid Ga corrosion has been observed.

It is notable that previous work has studied various TSMs and claimed that their topological surface states are robust against surface defect formation. For example, Liu *et al.* have reported that for the Dirac semimetal Na_3_Bi topological surface states can persist even after Na vacancies are introduced into the crystal structure.^56^ However, other theoretical work has shown that surface Fermi arcs in TSMs are not robust under disorder. Wilson *et al.* showed that when disorder is introduced, the surface states of Weyl semimetals will hybridize with the bulk states and therefore the Fermi arcs are not topologically protected from disorder.^57^ We would like to point out that in contrast to the approach used by Liu and coworkers, which uses an increment in the on-site energy of the orbitals of the atoms at the surface of the material to model the effect of Na vacancies, our approach of modeling slabs with vacancies attempts to capture the important effect of lattice reconstruction that is not included in their model. In fact, recent work studying electrocatalysis with topological materials have also found the dynamic reconstruction of catalyst surfaces to significantly adjust the surface topological states, echoing our results here.^32–34^ Furthermore, the defect size in experiments, as shown in Fig. 2, is well beyond the atomistic length scale and should involve even more drastic lattice reconstruction effects beyond what we can capture in our computational slab model. Therefore, the nanoporous, Pt-enriched phase that forms at the PtGa surface after CV cycling, which is dramatically different from its original structural form, is unlikely to have non-trivial topology.

Although the results presented herein focus on PtGa, other topological materials that have been reported as electrocatalysts may also require careful evaluation for possible corrosion or surface reconstruction in electrochemical environments. The expected stability of candidate topological semimetals can be screened using Pourbaix diagrams, which have been derived for many metals and metalloids on the periodic table and are conveniently collated in Marcel Pourbaix's Atlas of Electrochemical Equilibria in Aqueous Solutions.^43^ Since many TSMs are composed of oxidatively sensitive elements (Si, Sn, Al, P, Bi, *etc.*),^2,3,8,56^ further investigation of the structural stability of TSMs under electrochemical operating conditions is needed to clarify the origin of their exceptional electrocatalytic reactivity.

## Conclusion

We evaluated the stability of PtGa, a topological semimetal, under electrochemical cycling within the aqueous non-faradaic potential window and found that its surface is not robust. Under these conditions, Ga corrodes and leaches into solution, leaving a Pt-rich nanoporous layer at the top of the crystal, as shown by ICP-MS and STEM analyses. Corresponding HER activity measurements reveal that the electrocatalytic performance of PtGa is simply correlated with an increase in the number of Pt active sites following Ga corrosion. When the concentration of these active sites is rigorously quantified using electrochemical CO stripping, lower HER performance is observed for PtGa than for Pt. Ga corrosion is additionally observed under more reducing conditions. Although PtGa is more structurally robust under these conditions, our calculations show that PtGa's topologically non-trivial surface states are still affected by the structural changes that take place during electrochemical operation. Together, these findings indicate that the topological character of PtGa is unlikely to survive practical electrochemical operation. Ultimately, these results highlight the importance of detailed surface characterization and structural elucidation during and following catalysis when evaluating heterogeneous electrocatalysts. This analysis is critical for accurately benchmarking the intrinsic activities of candidate heterogeneous electrocatalysts.

More broadly, this work highlights the distinct meaning of robustness in chemistry and physics, as modeling simple perturbations to the surface of TSMs that are often considered in the condensed matter community, such as vacancies and adsorbed atoms, is insufficient for capturing the pH and potential dependent structural changes that take place on dynamic electrocatalytic reaction surfaces.

## Author contributions

All the authors contributed to the intellectual content of this project. B. J. carried out the electrochemical, ICP-MS, and XPS studies. M.-F. C. carried out the theoretical work and analysis. M. Z. performed the electron microscopy studies. P. G. synthesized the PtGa crystal and analyzed the single-crystal X-ray diffraction data. S. X., J. L, and D. G. N. conceived and supervised the project. All authors participated in the writing and editing of the manuscript. B. J., M.-F. C., M. Z.: these authors contributed equally and retain the right to list co-first authors in any order on personal documents such as curricula vitae.

## Conflicts of interest

The authors declare no competing interests.

## Supplementary Material

SC-017-D6SC03026B-s001

SC-017-D6SC03026B-s002

SC-017-D6SC03026B-s003

SC-017-D6SC03026B-s004

SC-017-D6SC03026B-s005

SC-017-D6SC03026B-s006

SC-017-D6SC03026B-s007

SC-017-D6SC03026B-s008

SC-017-D6SC03026B-s009

SC-017-D6SC03026B-s010

SC-017-D6SC03026B-s011

SC-017-D6SC03026B-s012

SC-017-D6SC03026B-s013

SC-017-D6SC03026B-s014

## Data Availability

CCDC 2505424 contains the supplementary crystallographic data for this paper.^58^ The data supporting this article have been included as part of the supplementary information (SI). Supplementary information is available. See DOI: https://doi.org/10.1039/d6sc03026b.
