# Supplementary figures and images for: Revisiting the origin of electrochemical activity in the topological semimetal PtGa
Source: Chem Sci. 2026 Jun 11;17(29):14359–66. doi: 10.1039/d6sc03026b (PMC13273368; doi:10.1039/d6sc03026b)

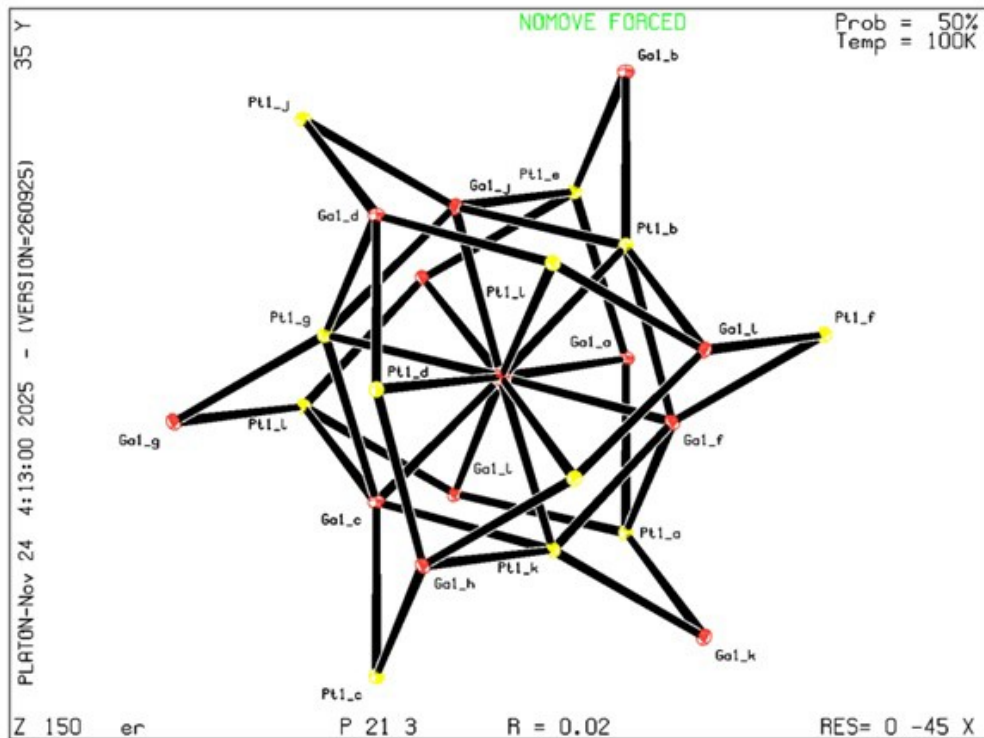

Supplement: SC-017-D6SC03026B-s001 [file SC-017-D6SC03026B-s001.pdf]

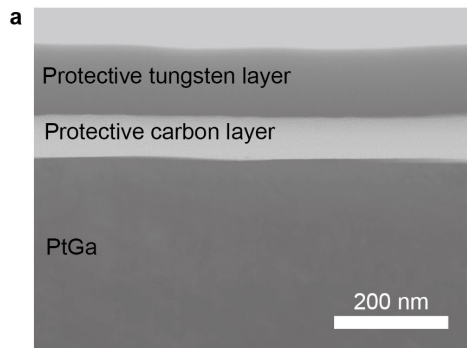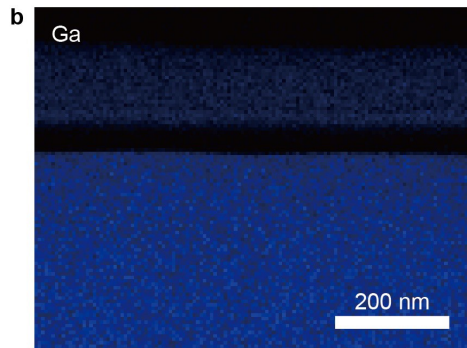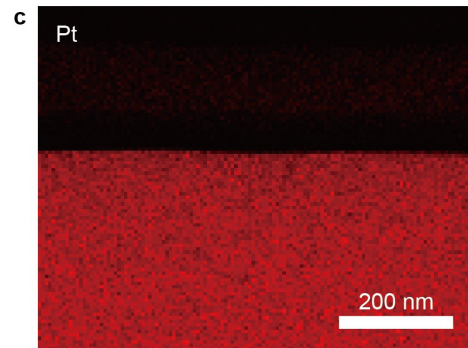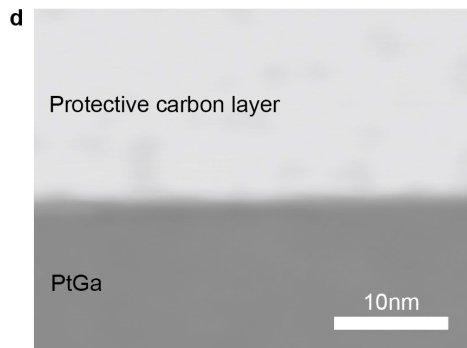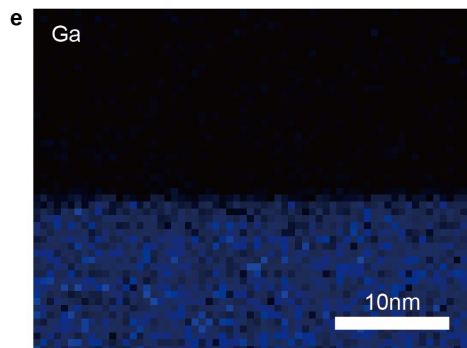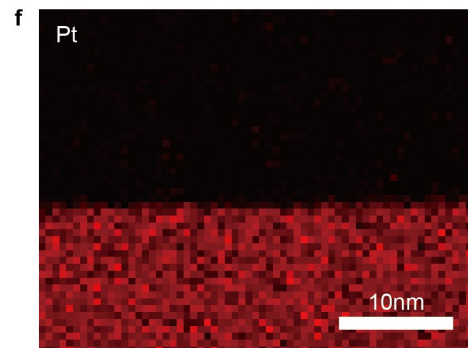

Supplement: SC-017-D6SC03026B-s002 [file SC-017-D6SC03026B-s002.pdf]

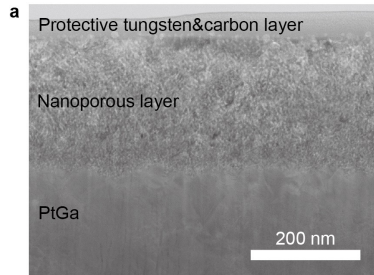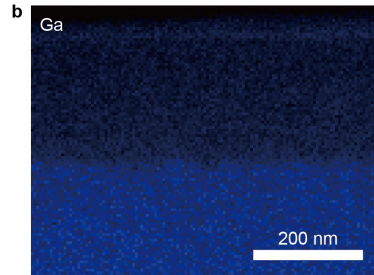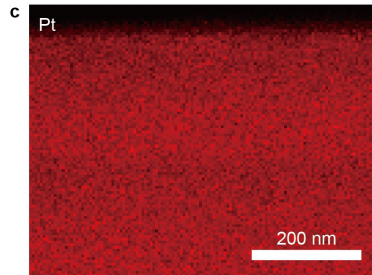

Supplement: SC-017-D6SC03026B-s003 [file SC-017-D6SC03026B-s003.pdf]

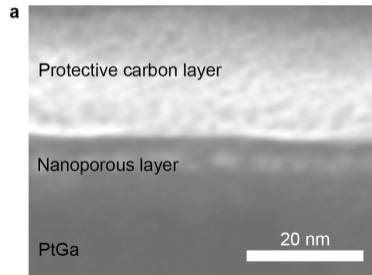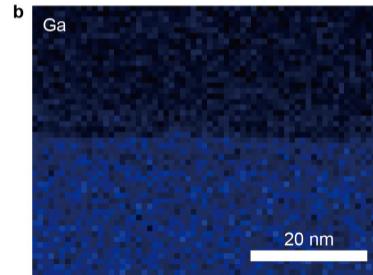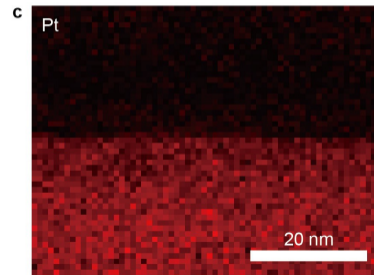

Supplement: SC-017-D6SC03026B-s004 [file SC-017-D6SC03026B-s004.pdf]

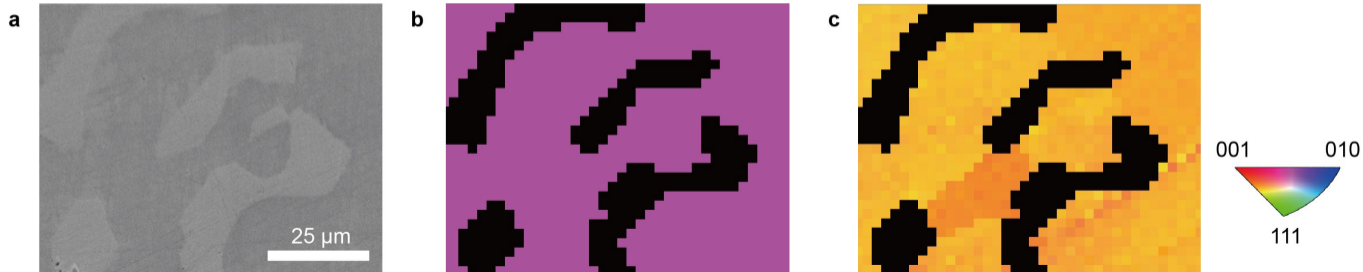

Supplement: SC-017-D6SC03026B-s007 [file SC-017-D6SC03026B-s007.pdf]

**PtGa(100)**

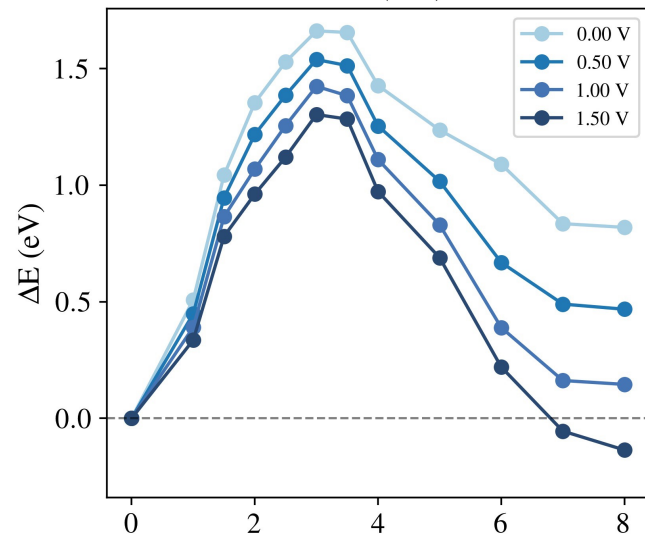

**PtGa(110)**

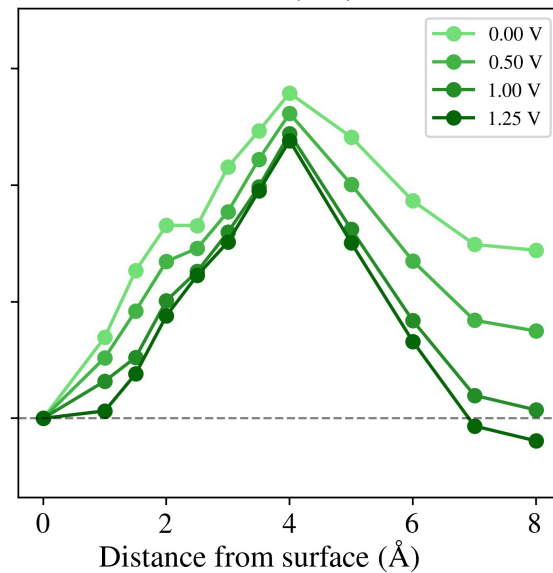

**PtGa(111)**

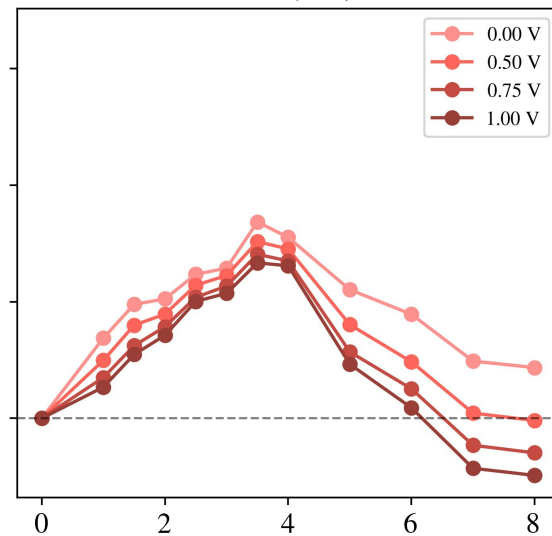

Supplement: SC-017-D6SC03026B-s008 [file SC-017-D6SC03026B-s008.pdf]

**PtGa(100) / PBE**

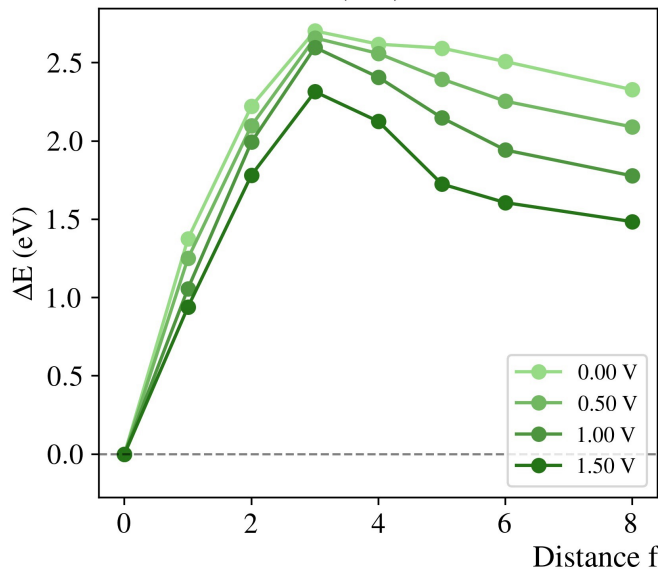

**PtGa(100) / RPBE**

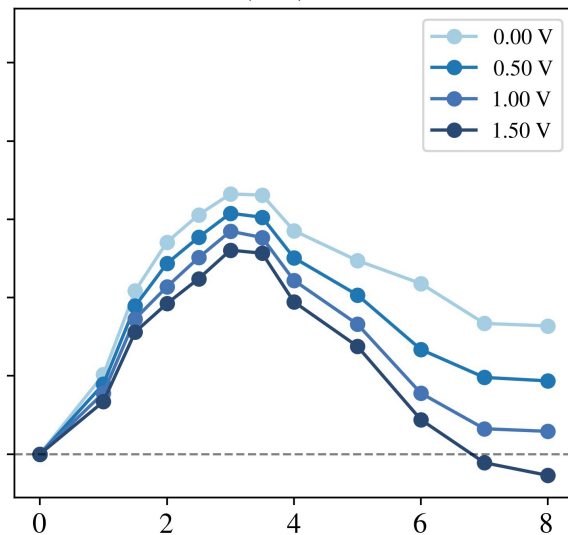

Supplement: SC-017-D6SC03026B-s009 [file SC-017-D6SC03026B-s009.pdf]

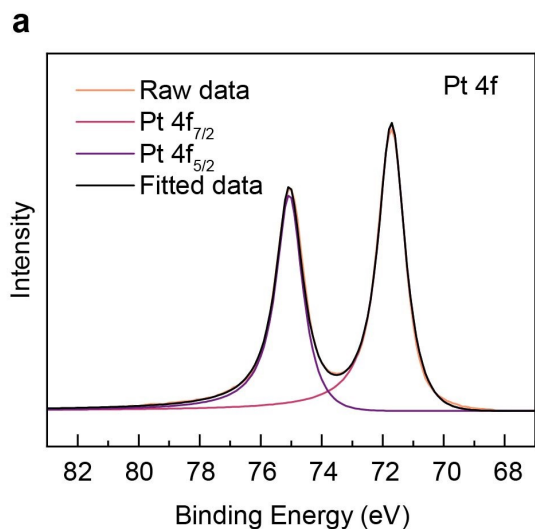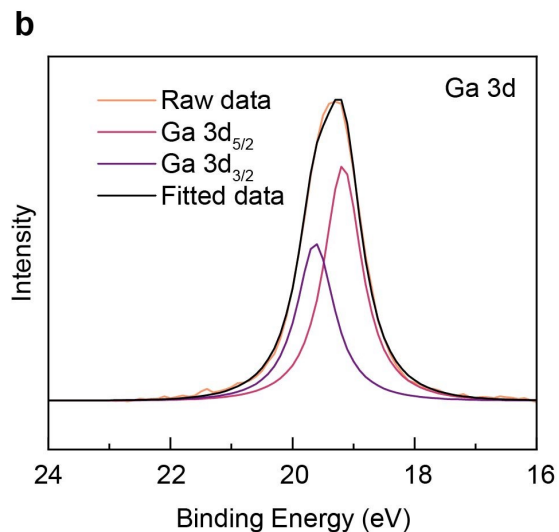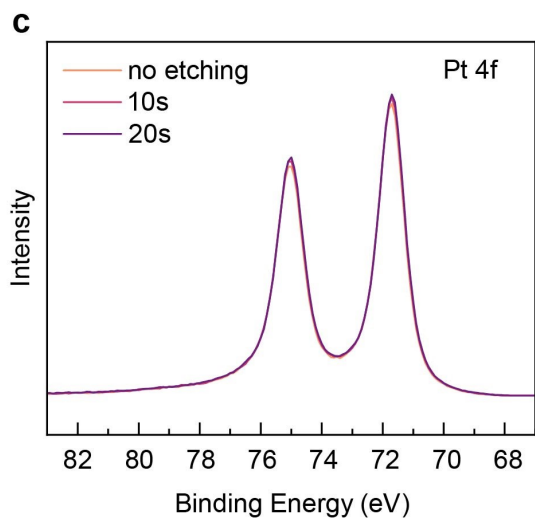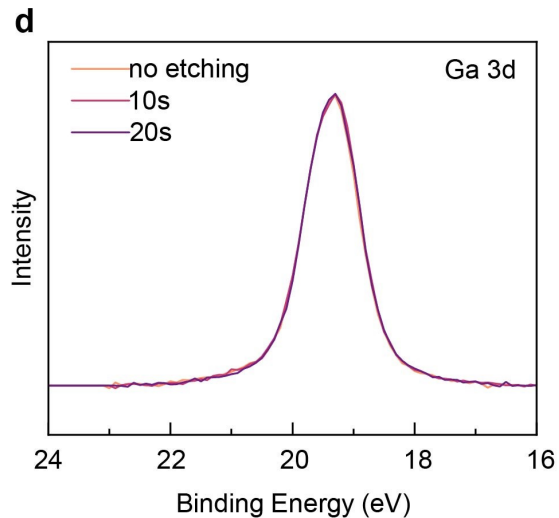

Supplement: SC-017-D6SC03026B-s010 [file SC-017-D6SC03026B-s010.pdf]

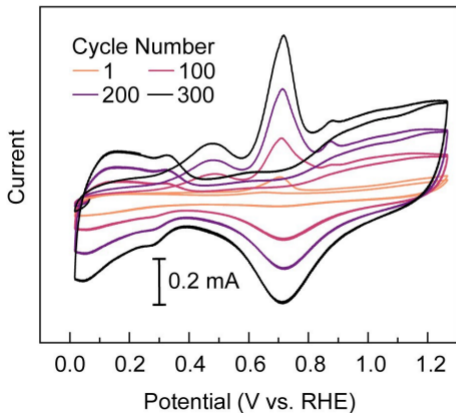

Supplement: SC-017-D6SC03026B-s011 [file SC-017-D6SC03026B-s011.pdf]

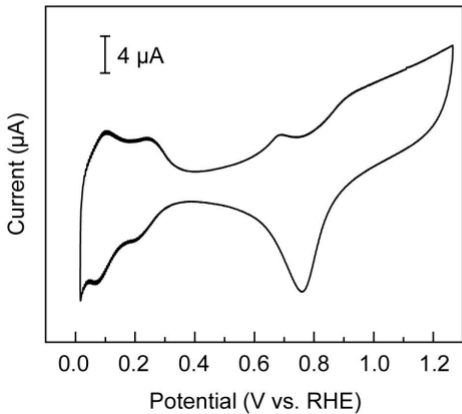

Supplement: SC-017-D6SC03026B-s012 [file SC-017-D6SC03026B-s012.pdf]

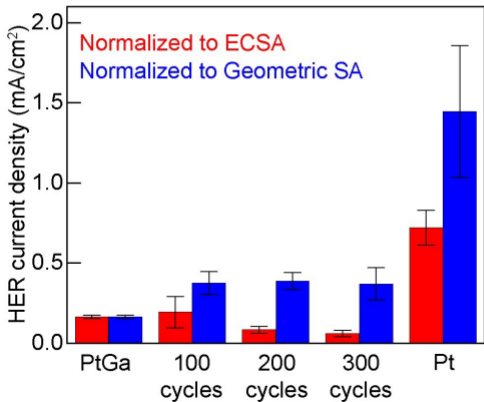

Supplement: SC-017-D6SC03026B-s013 [file SC-017-D6SC03026B-s013.pdf]

**a**

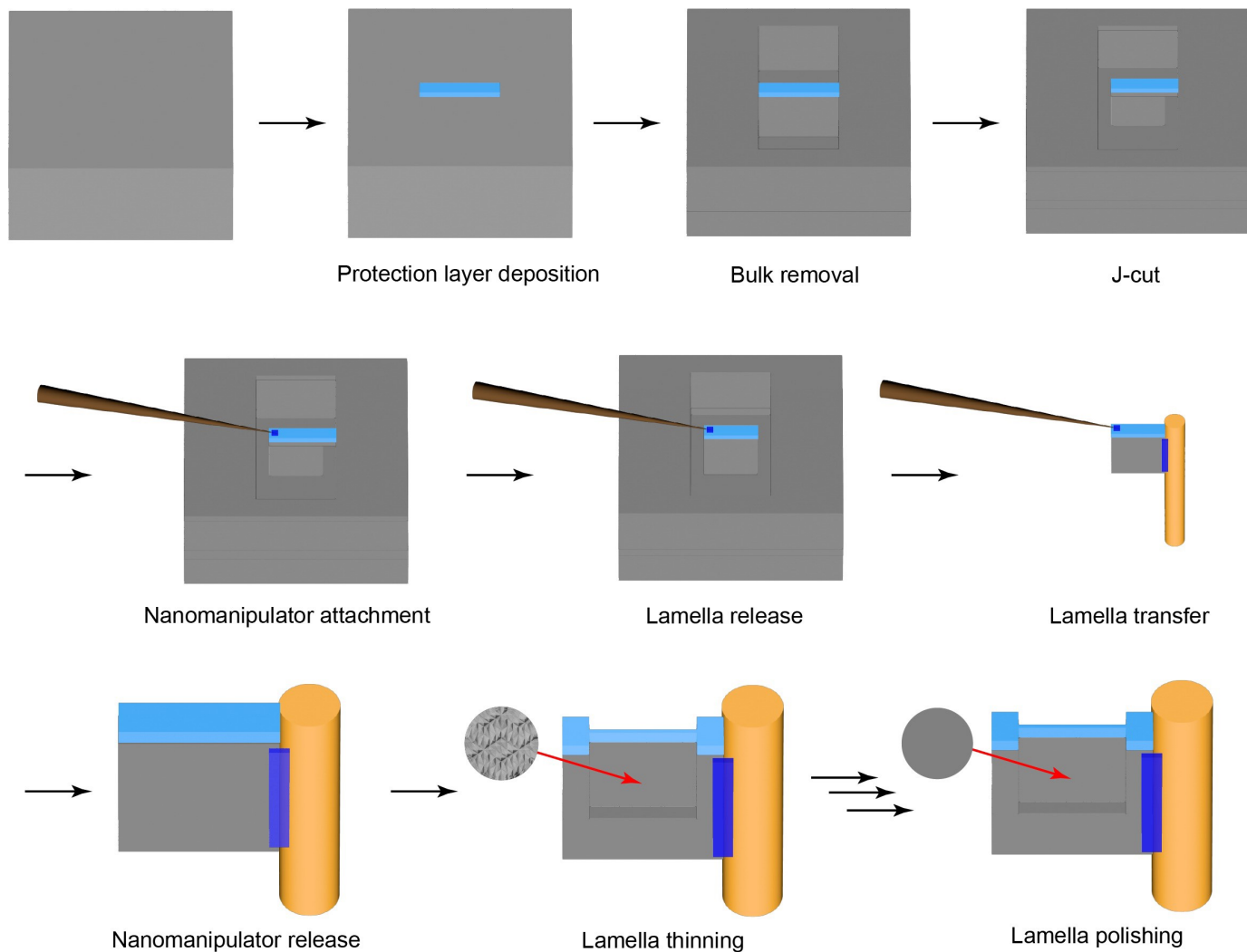

**b**

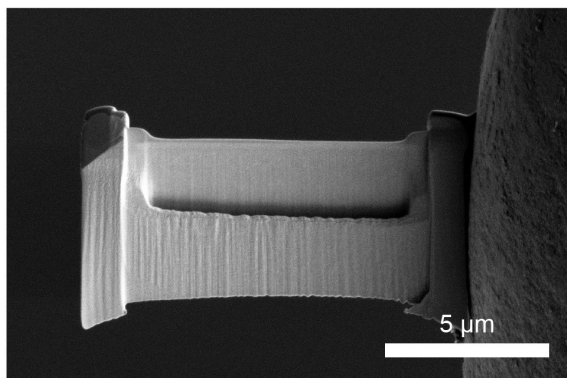

Supplement: SC-017-D6SC03026B-s014 [file SC-017-D6SC03026B-s014.pdf]
