# Supplementary material for: Revisiting the origin of electrochemical activity in the topological semimetal PtGa
Source: Chem Sci. 2026 Jun 11;17(29):14359–66. doi: 10.1039/d6sc03026b (PMC13273368; doi:10.1039/d6sc03026b)
Supplement: SC-017-D6SC03026B-s005 [file SC-017-D6SC03026B-s005.pdf]

Supplementary Information (SI) for Chemical Science.
This journal is © The Royal Society of Chemistry 2026

Supplementary Information for: Revisiting the Origin of Electrochemical Activity in the Topological Semimetal PtGa

Brandon Johnston*[a], Meng-Fu Chen*[a], Mingxuan Zhu*[a], Peng Guo[a], Su-Yang Xu†[a], Joonho Lee‡[a], Daniel G. Nocera¶[a]

[a] *Department of Chemistry and Chemical Biology, Harvard University, 12 Oxford St., Cambridge 02138, MA, USA*

* These authors contributed equally to this work and are listed in alphabetical order.

† Email: suyangxu@fas.harvard.edu.

‡ Email: joonholee@g.harvard.edu.

¶ Email: dnocera@fas.harvard.edu.

Single Crystal X-ray Diffraction

Single-crystal X-ray diffraction (SCXRD) measurements were performed on PtGa crystals coated with Paratone-N oil and mounted on MiTeGen loops. SCXRD data were collected at the Harvard University Department of Chemistry and Chemical Biology X-ray Laboratory (X-ray Core) using Mo Kα radiation on an APEX DUO single crystal diffractometer equipped with an APEX II CCD detector. Crystals were frozen at near 100 K during data collection using an Oxford Cryosystems Cryostream. Raw data were integrated and corrected for Lorentz and polarization effects using Bruker AXS SAINT software and corrected for absorption using SADABS.[1] The space group assignment was determined by examining a variety of factors, including systematic absences and E-statistics, as well as successive refinement of the structures. Structures were solved by intrinsic phasing using SHELXT and refined using SHELXL operated in the OLEX2 interface.[2–4] Further information on refinement strategies is provided in the crystallographic information file (CCDC deposition number 2505424). Additional crystallographic data are provided in Table S1.

Table S1: Crystallographic data for PtGa.

Compound	PtGa
Crystal system	Cubic
Space group	$P2_13$ (No. 198)
Formula weight	264.81
$a = b = c$ (Å)	4.8979(3)
$\alpha = \beta = \gamma$ (°)	90
Cell volume (Å^3)	117.50(2)
Z	4
Density (g/cm^3)	14.970
Radiation / wavelength (Å)	Mo Kα, 0.71073
Measured fraction $\theta_{\max}$	25.028
Reflections number	708
R_{int}	0.0503
Parameters refined	7
R_1 ($I > 2\sigma(I)$)	0.0200
wR_2 (all data)	0.0409
Goodness-of-fit (GOF)	1.360
Largest diff. peak (e Å^{-3})	1.277
Largest diff. hole (e Å^{-3})	−1.981

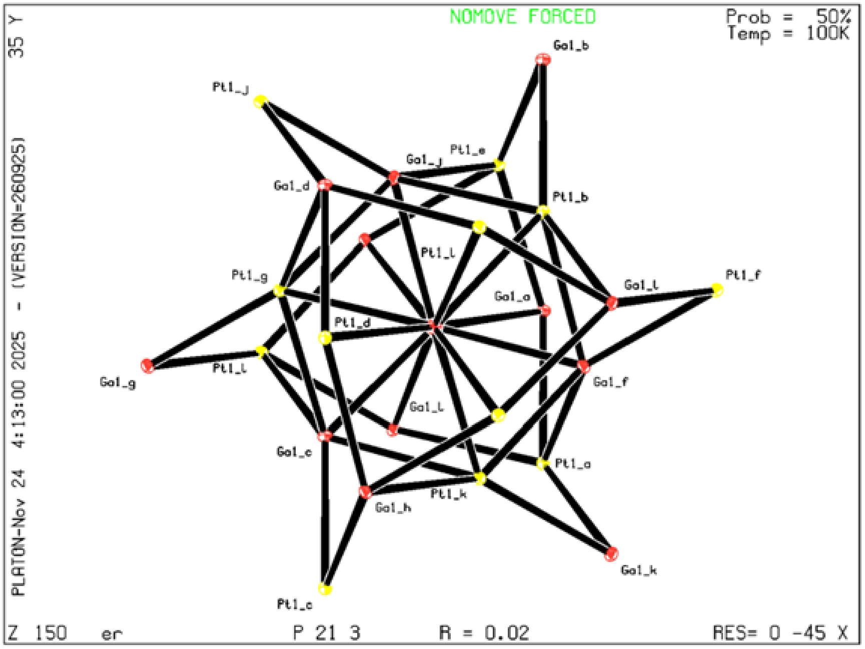

Figure S1: Crystal structure of compound PtGa as generated from the checkcif analysis.

X-ray Photoelectron Spectroscopy

XPS measurements were performed using a Thermo Scientific KAlpha+ XPS system equipped with a monochromatic soft aluminum X-ray (1.4866 keV, line width $\approx$ 0.3 eV) generated by a 12 kV electron beam. The instrument is additionally equipped with a 180° double-focusing hemispherical analyzer and 128-channel detector. A 400 μM $\times$ 400 μM spot size was used for all measurements, and surface charge compensation was accomplished by using a low energy electron flood gun. High resolution spectra were collected for the Pt 4f, Ga 3d, O 1s, and C 1s regions by averaging 5–10 scans (step size = 0.1 eV). Multiple spots on each sample were collected to ensure sample uniformity. All spectra were normalized and calibrated using the adventitious carbon peak at 284.8 eV. Pt 4f peaks were fit using a hybrid Doniach–Sunjic/Gaussian–Lorentzian line shape, and the Pt $4f_{7/2}$ and Pt $4f_{5/2}$ transitions were constrained to a 3.35 eV energy separation.[5] Ga 3d peaks were fit using a Gaussian–Lorentzian line shape, and the Ga $3d_{5/2}$ and Ga $3d_{3/2}$ transitions were constrained to a 0.449 eV energy separation.[6] Depth profiles were collected by sputtering the sample surface with a monatomic Ar^+ ion source (4000 eV) between measurements.

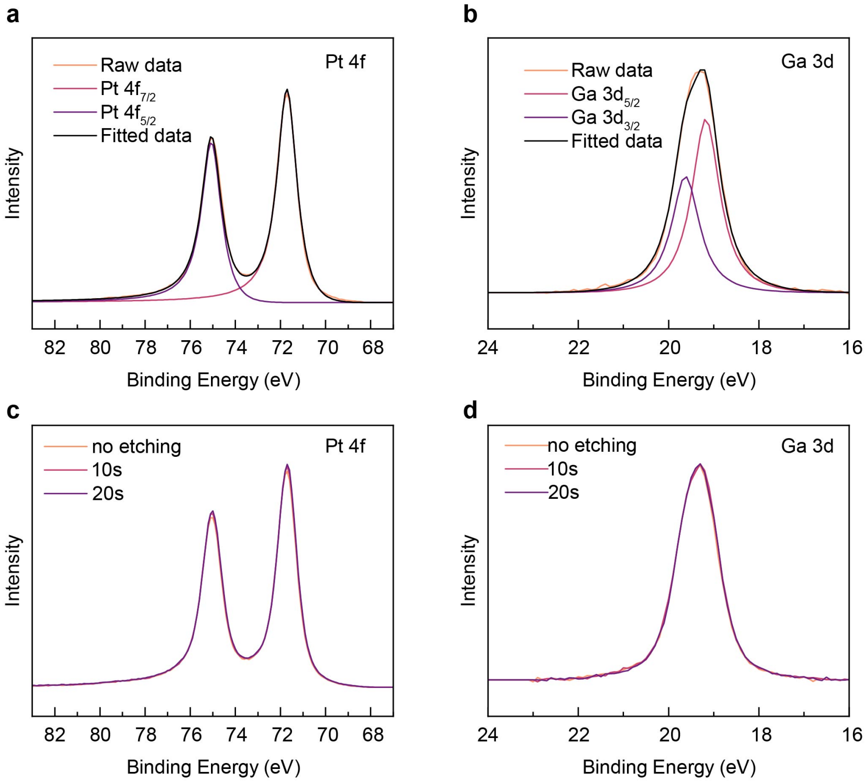

Figure S2: **a** Pt 4f XPS spectra of PtGa (orange). The red and purple traces indicate fits for the Pt $4f_{7/2}$ and Pt $4f_{5/2}$ transitions, respectively. The total fit is indicated by the black trace. **b** Ga 3d XPS spectra of PtGa (orange). The red and purple traces indicate fits for the Ga $3d_{5/2}$ and Ga $3d_{3/2}$ transitions, respectively. The total fit is indicated by the black trace. **c** Pt 4f XPS spectra and **d** Ga 3d XPS spectra of PtGa before and after Ar sputter cleaning.

Inductively-coupled Plasma Mass Spectrometry

Solution nebulization ICP-MS measurements were performed using a Thermo Electron X-Series quadrupole ICP-MS instrument. Sample aliquots were taken directly from the electrolyte solution after the indicated number of cyclic voltammetry cycles and subsequently diluted in 2% HNO_3 (TraceMetal^{TM}) prior to analysis. Standard solutions were prepared by diluting TraceCert Pt and Ga analytical standards in 2% HNO_3 solution. Sample Ga and Pt concentrations were calculated from an instrument calibration curve derived from standard solutions. A known amount of TraceCert Yttrium analytical standard was added to each sample to correct for instrument drift.

Electrochemistry

All electrochemical experiments were recorded with a CH Instruments 660C or 760E electrochemical workstation using a three-electrode cell with a leak-free Ag/AgCl reference electrode, a Pt mesh counter electrode, and a PtGa working electrode. The PtGa working electrode was constructed by clipping the PtGa crystal directly to a stainless-steel alligator clip and submerging the crystal in the electrolyte solution. A fresh surface was exposed by polishing the PtGa crystal using fine-grit sandpaper prior to recording each electrochemical experiment. During electrochemical experiments, multiple crystal facets were immersed in the solution. All experiments were conducted in 0.1 M $HClO_4$. Electrode potentials were converted to the RHE scale using $E(\text{RHE}) = 0.196 + E(\text{Ag/AgCl}) + 0.059 \times \text{pH}$. ECSA measurements were performed using an electrochemical CO stripping technique, as described previously.[7]

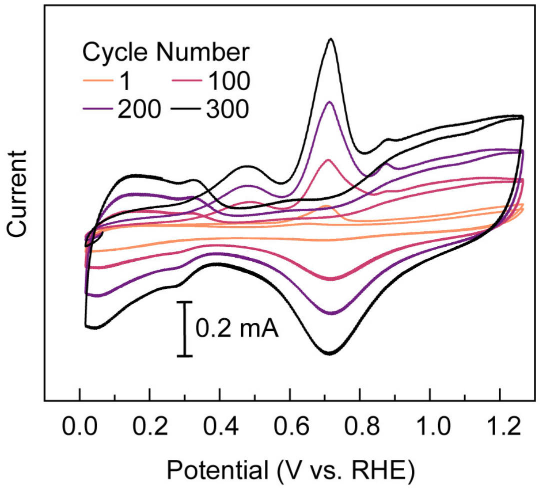

Figure S3: Electrochemical CO stripping curves of PtGa in 0.1 M $HClO_4$ following electrochemical cycling in the non-faradaic region (scan rate = 50 mV/s).

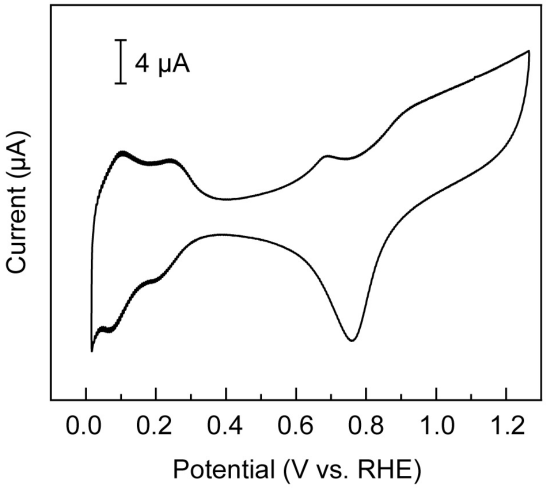

Figure S4: Cyclic voltammogram of Pt disk in 0.1 M $HClO_4$ in the non-faradaic region (scan rate = 50 mV/s).

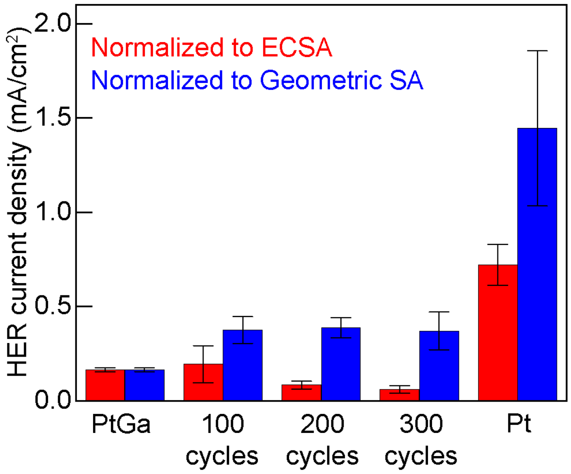

Figure S5: HER activity of PtGa before and after electrochemically cycling between 0 V and 1.2 V vs. RHE in 0.1 M $HClO_4$ (scan rate = 50 mV/s). HER activity measurements were performed in triplicate after the indicated number of cycles by polarizing the electrode at −0.02 V vs. RHE and recording the steady-state current response. The ECSA was determined using CO stripping voltammetry by following a previously reported procedure.[7] Since the geometric surface area of PtGa could not be determined, it was taken to be equal to the initial ECSA. The HER activity of a Pt disk is included for comparison.

Electron Microscopy Imaging

Lamellae of the PtGa crystal surface were prepared[8] using an FEI Helios NanoLab 660 DualBeam system. First, protective carbon and tungsten layers were deposited on the sample surface using an electron beam and a Ga ion beam, respectively. Subsequently, a 2 μm-thick lamella was milled with a Ga ion beam (30 kV, 2.5 nA), lifted out, and mounted on a TEM half-grid. The lamella was further thinned to around 150 nm by a Ga ion beam (30 kV, 80 pA). Finally, the lamella was polished to around 80 nm with a final round of Ga ion milling (5 kV, 41 pA) (Fig. S6). Ga implantation at the lamella surface was removed with a Fischione Model 1040 Nanomill. Each side of the lamella was further polished with an Ar ion beam (700 V, 200 μA) for 2 min,[9] removing 6 nm of material. Imaging was performed on a JEOL ARM 200F STEM at 200 kV. The ADF images were collected at 180 mrad. EDS analysis was performed on an EDAX Octane 100 mm^2 detector integrated with the microscope. EBSD imaging[10] was performed on an Oxford Symmetry EBSD system integrated with a JEOL 7900F SEM.

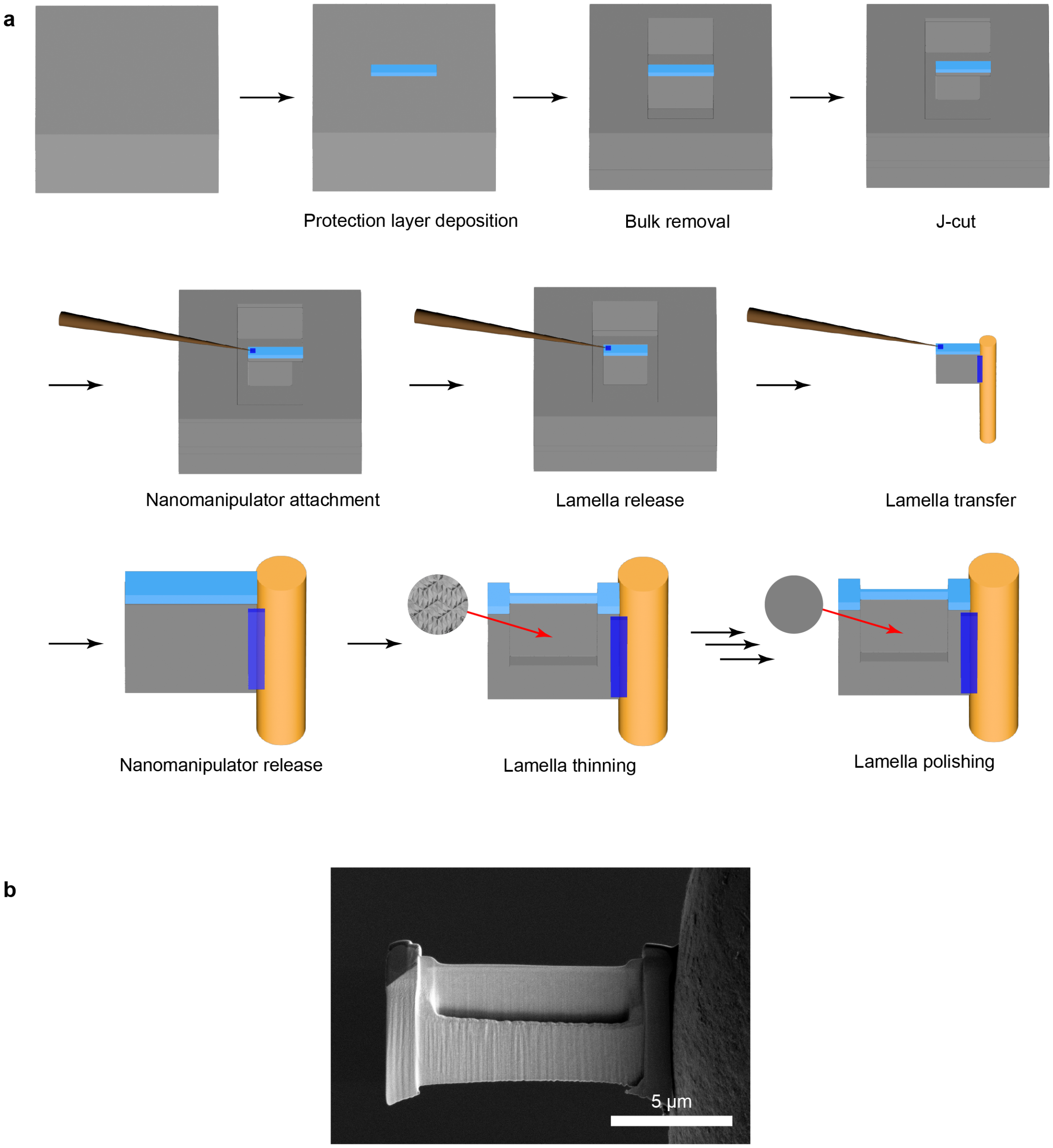

Figure S6: **a** Scheme of PtGa lamella preparation. **b** SEM image of a representative PtGa lamella prepared by FIB.

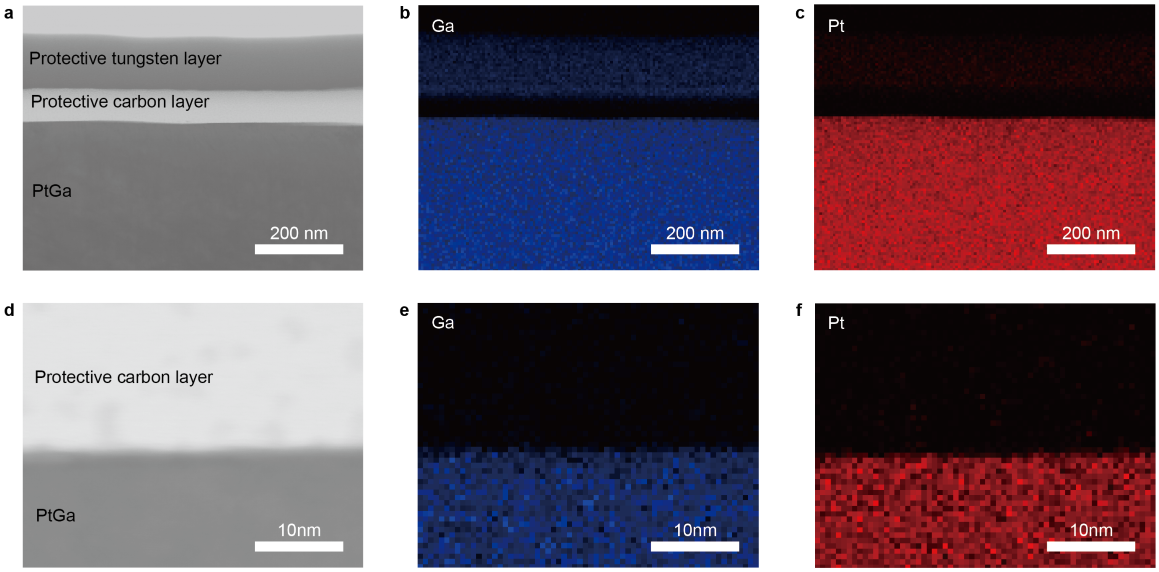

Figure S7: **a, d** Bright field (BF)-STEM images of PtGa lamella without any electrochemical treatment. **b, e** EDS mapping of Ga. **c, f** EDS mapping of Pt.

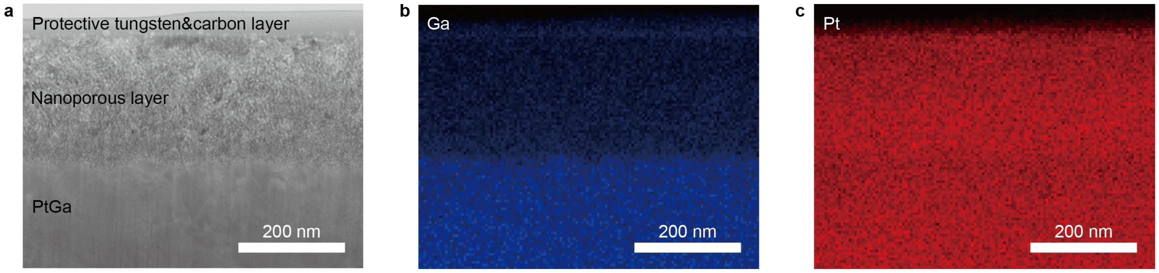

Figure S8: **a** BF-STEM images of PtGa lamella after 800 CV cycles between 0.0 V and 1.2 V vs RHE. **b** EDS mapping of Ga. **c** EDS mapping of Pt.

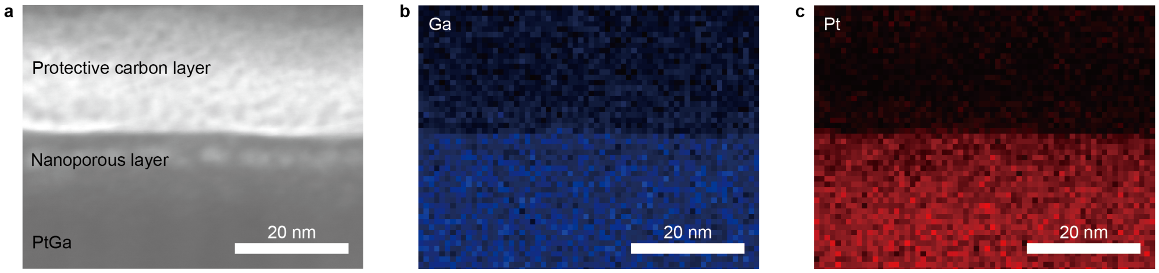

Figure S9: **a** BF-STEM images of PtGa lamella after polarization at −0.09 V vs. RHE. **b** EDS mapping of Ga. **c** EDS mapping of Pt.

Electron Backscatter Diffraction (EBSD)

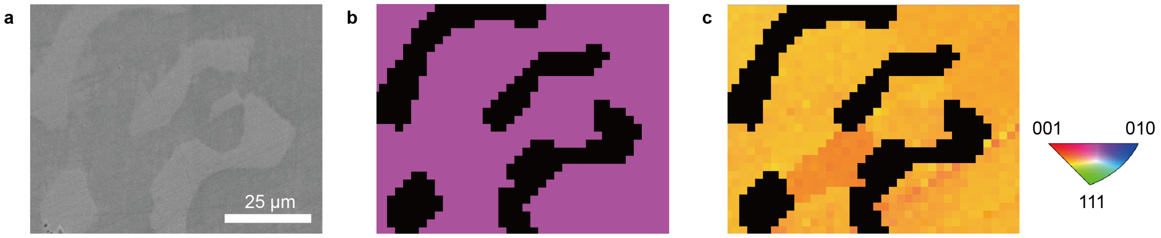

Figure S10: **a** SEM image of freshly polished PtGa. **b** EBSD phase map. Purple region indexes to the PtGa crystal structure. **c** EBSD orientation map.

Expected Corrosion Calculations

After 300 cycles, the nanoporous leaching layer would be expected to extend 93 nm below the surface (0.31 nm/cycle × 300 cycles). Using the initial ECSA before electrochemical operation (0.36 cm^2), the total expected volume of the nanoporous leaching layer can be calculated as follows:

$$V_{\text{layer}} = (9.3 \times 10^{-6}\ \text{cm}) \times (0.36\ \text{cm}^2) = 3.3 \times 10^{-6}\ \text{cm}^3$$

The volume of a single PtGa unit cell can be calculated using the lattice constant:

$$V_{\text{u.c.}} = (4.9 \times 10^{-8}\ \text{cm})^3 = 1.2 \times 10^{-22}\ \text{cm}^3$$

The expected number of unit cells in the leaching layer is:

$$\frac{V_{\text{layer}}}{V_{\text{u.c.}}} = 2.9 \times 10^{16}$$

Each unit cell initially contains 4 Pt atoms and 4 Ga atoms, but the stoichiometry of the nanoporous leaching layer is $Pt_{2.6}Ga$. Assuming Pt dissolution is negligible, the number of Ga atoms that leach from each unit cell is:

$$4 - \frac{4}{2.6} = 2.4$$

Thus, the total number of moles of Ga that would be expected to leach from the surface would be

$$2.9 \times 10^{16} \times 2.4 \times \frac{\text{mol}}{6.022 \times 10^{23}} = 1.2 \times 10^{-7}\ \text{mol}$$

Computational Details

Band Structure Calculations. Band structure calculations were carried out using Quantum Espresso.[11,12] The Perdew–Burke–Ernzerhof (PBE) functional was used along with the projector augmented wave method and spin-orbit coupling.[13,14] For the surface model, we chose a 2×1 slab with a thickness of 5 unit cells, which has been shown to be thick enough for capturing bulk-like characteristics in previous works.[15] A vacuum layer of ≈ 25 on the z-direction was included to prevent spurious interactions between neighboring unit cells. To simulate the Ga defect formation observed in experiments, we removed Ga atoms from the top layer of the PtGa slab and relaxed the geometry of the atoms in the top two layers of the slab model. SCF and NSCF calculations with spin-orbit coupling were then carried out to obtain the electronic structure of the slab with an $8 \times 8 \times 1$ Monkhorst–Pack k-point mesh. A kinetic cutoff of 60 Rydberg is used for the SCF calculations. To plot the surface band structure, we constructed the tight-binding (TB) Hamiltonian using Maximally Localized Wannier Functions (MLWFs) from Wannier90[16] with projections from the d orbitals of Pt and the p orbitals of Ga.

Ga Atom Dissociation Calculations. For the Ga atom dissolution calculations, we used a five-layer 2×2 PtGa slab and displaced the Ga atom at the surface with the least coordination away from its initial position into the vacuum layer. DFT calculations were carried out using the revised PBE (RPBE) density functional[17] combined with projector augmented wave pseudopotentials (PAW)[13,18] as implemented in the Vienna ab initio simulation package (VASP) 5.4.4. The energy cutoff used for the plane wave was 500 eV. Second-order smearing via the method of Methfessel–Paxton[19] was used with a smearing width of 0.1 eV. To emulate the solvation environment found in experimental electrochemical cells, we used an implicit solvation model with six additional explicit water molecules to solvate the dissociated Ga atom, reflecting the hexacoordinated Gallium water complex. We used the VASPsol++ package to perform grand-canonical DFT simulations with the nonlinear, nonlocal polarizable continuum model for implicit solvation.[20]

Grand Canonical DFT calculations for Ga corrosion process

We present additional GC-DFT calculations of the Ga dissolution process. In Fig. S11, we show a large facet dependence for the onset potential of thermodynamically favorable dissolution, as well as the kinetic barrier of dissolution at different oxidative potentials. However, the onset potential for thermodynamically favorable dissolution is significantly higher than the experimentally measured value for all facets, and the kinetic barrier is too high for this process to be a rapid corrosion reaction. This is likely due to the inherent limitations of our model in capturing the full picture of the dissolution process; in particular, we can include only 6 solvent molecules in our explicit solvation model because of the large degrees of freedom required to obtain reasonable geometries. Realistically, both the Ga atom and the PtGa slab surface should be consistently solvated by water molecules throughout the dissolution process, thereby stabilizing the dissolved structure and significantly decreasing both the onset potential for thermodynamically favorable dissolution and the kinetic barrier to the process.

Further on, in Fig. S12 we show the large variation in the calculated dissociation profile depending on the selected density functional. Even for functionals on the same rung of Jacob's ladder and from the same family (PBE versus RPBE), the predicted kinetic barrier can be different by as large as 1 eV. This calls for higher-level theoretical methods that can work in the grand-canonical framework and resolve the ambiguity of pure functionals, a research direction that some of us are actively pursuing.

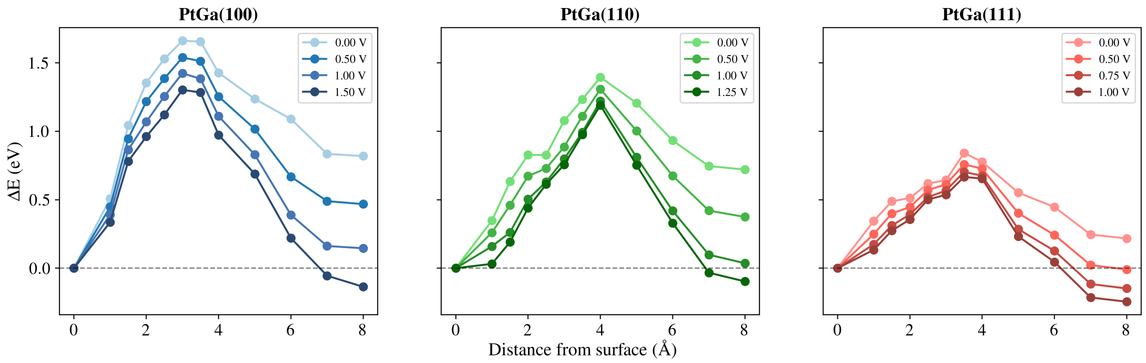

Figure S11: Facet dependence of the thermodynamics and kinetic barrier of Ga atom dissolution.

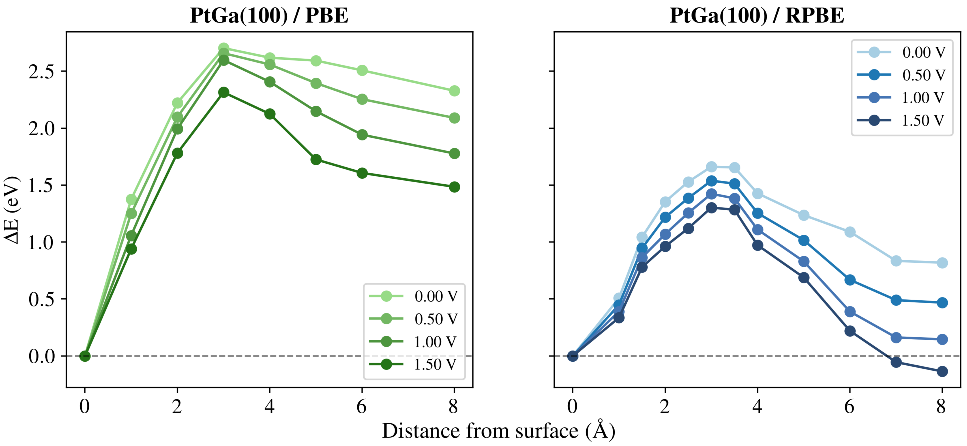

Figure S12: Functional dependence of the energetics of Ga atom dissolution.

References

[1] G. M. Sheldrick, *SADABS*, Bruker Analytical X-ray Systems, 2014.

[2] G. M. Sheldrick, *Acta Crystallographica Section A*, 2015, **71**, 3–8.

[3] G. M. Sheldrick, *Acta Crystallographica Section C*, 2015, **71**, 3–8.

[4] O. V. Dolomanov, L. J. Bourhis, R. J. Gildea, J. A. K. Howard and H. Puschmann, *Journal of Applied Crystallography*, 2009, **42**, 339–341.

[5] M.-J. Choi, H. Park, M. H. Engelhard, D. Li, P. V. Sushko and Y. Du, *Journal of Vacuum Science & Technology A*, 2024, **42**, 063209.

[6] J. L. Bourque, M. C. Biesinger and K. M. Baines, *Dalton Transactions*, 2016, **45**, 7678–7696.

[7] T. Binninger, E. Fabbri, R. Kötz and T. J. Schmidt, *Journal of The Electrochemical Society*, 2013, **161**, H121–H128.

[8] J. Mayer, L. A. Giannuzzi, T. Kamino and J. Michael, *MRS Bulletin*, 2007, **32**, 400–407.

[9] T. Sato, Y. Aizawa, H. Matsumoto, M. Kiyohara, C. Kamiya and F. Von Cube, *Journal of Microscopy*, 2020, **279**, 234–241.

[10] A. J. Wilkinson and T. B. Britton, *Materials Today*, 2012, **15**, 366–376.

[11] P. Giannozzi, S. Baroni, N. Bonini, M. Calandra, R. Car, C. Cavazzoni, D. Ceresoli, G. L. Chiarotti, M. Cococcioni, I. Dabo, A. Dal Corso, S. de Gironcoli, S. Fabris, G. Fratesi, R. Gebauer, U. Gerstmann, C. Gougoussis, A. Kokalj, M. Lazzeri, L. Martin-Samos, N. Marzari, F. Mauri, R. Mazzarello, S. Paolini, A. Pasquarello, L. Paulatto, C. Sbraccia, S. Scandolo, G. Sclauzero, A. P. Seitsonen, A. Smogunov, P. Umari and R. M. Wentzcovitch, *Journal of Physics: Condensed Matter*, 2009, **21**, 395502.

[12] P. Giannozzi, O. Andreussi, T. Brumme, O. Bunau, M. Buongiorno Nardelli, M. Calandra, R. Car, C. Cavazzoni, D. Ceresoli, M. Cococcioni, N. Colonna, I. Carnimeo, A. Dal Corso, S. de Gironcoli, P. Delugas, R. A. DiStasio, A. Ferretti, A. Floris, G. Fratesi, G. Fugallo, R. Gebauer, U. Gerstmann, F. Giustino, T. Gorni, J. Jia, M. Kawamura, H.-Y. Ko, A. Kokalj, E. Küçükbenli, M. Lazzeri, M. Marsili, N. Marzari, F. Mauri, N. L. Nguyen, H.-V. Nguyen, A. Otero-de-la Roza, L. Paulatto, S. Poncé, D. Rocca, R. Sabatini, B. Santra, M. Schlipf, A. P. Seitsonen, A. Smogunov, I. Timrov, T. Thonhauser, P. Umari, N. Vast, X. Wu and S. Baroni, *Journal of Physics: Condensed Matter*, 2017, **29**, 465901.

[13] P. E. Blöchl, *Physical Review B*, 1994, **50**, 17953–17979.

[14] A. Dal Corso, *Computational Materials Science*, 2014, **95**, 337–350.

[15] Q. Yang, G. Li, K. Manna, F. Fan, C. Felser and Y. Sun, *Advanced Materials*, 2020, **32**, 1908518.

[16] G. Pizzi, V. Vitale, R. Arita, S. Blügel, F. Freimuth, G. Géranton, M. Gibertini, D. Gresch, C. Johnson, T. Koretsune, J. Ibañez-Azpiroz, H. Lee, J.-M. Lihm, D. Marchand, A. Marrazzo, Y. Mokrousov, J. I. Mustafa, Y. Nohara, Y. Nomura, L. Paulatto, S. Poncé, T. Ponweiser, J. Qiao, F. Thöle, S. S. Tsirkin, M. Wierzbowska, N. Marzari, D. Vanderbilt, I. Souza, A. A. Mostofi and J. R. Yates, *Journal of Physics: Condensed Matter*, 2020, **32**, 165902.

[17] B. Hammer, L. B. Hansen and J. K. Nørskov, *Physical Review B*, 1999, **59**, 7413–7421.

[18] G. Kresse and D. Joubert, *Physical Review B*, 1999, **59**, 1758–1775.

[19] M. Methfessel and A. T. Paxton, *Physical Review B*, 1989, **40**, 3616–3621.

[20] S. M. R. Islam, F. Khezeli, S. Ringe and C. Plaisance, *The Journal of Chemical Physics*, 2023, **159**, 234117.
